# Turning on the Radio: Epigenetic Inhibitors as Potential Radiopriming Agents

**DOI:** 10.3390/biom6030032

**Published:** 2016-07-04

**Authors:** Bryan Oronsky, Jan Scicinski, Michelle M. Kim, Pedro Cabrales, Michael E. Salacz, Corey A. Carter, Neil Oronsky, Harry Lybeck, Michelle Lybeck, Christopher Larson, Tony R. Reid, Arnold Oronsky

**Affiliations:** 1 EpicentRx, Inc, Mountain View, CA 94040, USA; boronsky@epicentrx.com (B.O.); lybeck.michelle@gmail.com (M.L.); 2 Department of Radiation Oncology, University of Michigan, Ann Arbor, MI 48109, USA; michekim@med.umich.edu; 3 Department of Bioengineering UCSD, La Jolla, San Diego, CA 92093, USA; pcabrales@ucsd.edu; 4 KU Medical Center, Kansas University, Kansas City, KS 66160, USA; msalacz@kumc.edu; 5 Murtha Cancer Center, Walter Reed National Military Medical Center, Bethesda, MD 20889, USA; corey.carter33@gmail.com; 6 CFLS Data, Mountain View, CA 94040, USA; nco@cflsdata.com; 7 Department of Physiology, Helsinki University, 00100 Helsinki, Finland; harrylybeck@gmail.com; 8 Moores Cancer Center, UCSD, La Jolla, CA 92093, USA; chris.larson.mail@gmail.com (C.L.); tonyreid@ucsd.edu (T.R.R.); 9 InterWest Partners, Menlo Park, CA 94025, USA; AOronsky@interwest.com

**Keywords:** radiotherapy, radiosensitization, epigenetics, DNA methyltransferase inhibition, histone deacetylase inhibition, epigenetic priming, reactive oxygen species (ROS)

## Abstract

First introduced during the late 1800s, radiation therapy is fundamental to the treatment of cancer. In developed countries, approximately 60% of all patients receive radiation therapy (also known as the sixty percenters), which makes radioresistance in cancer an important and, to date, unsolved, clinical problem. Unfortunately, the therapeutic refractoriness of solid tumors is the rule not the exception, and the ubiquity of resistance also extends to standard chemotherapy, molecularly targeted therapy and immunotherapy. Based on extrapolation from recent clinical inroads with epigenetic agents to prime refractory tumors for maximum sensitivity to concurrent or subsequent therapies, the radioresistant phenotype is potentially reversible, since aberrant epigenetic mechanisms are critical contributors to the evolution of resistant subpopulations of malignant cells. Within the framework of a syllogism, this review explores the emerging link between epigenetics and the development of radioresistance and makes the case that a strategy of pre- or co-treatment with epigenetic agents has the potential to, not only derepress inappropriately silenced genes, but also increase reactive oxygen species production, resulting in the restoration of radiosensitivity.

## 1. Introduction

*All Men Are Mortal. Socrates Is a Man. Therefore, All Men Are* *Socrates*

—*Woody Allen*

In 1896, the same year that W.C. Roentgen discovered X-rays, the Chicagoan, Dr. Emil Grubbe, became the world’s first radiation oncologist when he treated a recurrent breast cancer patient with radiotherapy (XRT) [1,2]. Since that fateful year, the clinical armamentarium has relied heavily on the incorporation of ionizing radiation to treat cancer, albeit with significant modifications from the initial prescription in the early 1900s to treat until skin erythema or skin burn [3]. In developed countries, radiation therapy (RT) is one of the most frequently used therapeutic modalities with approximately 60% of all cancer patients receiving it in the course of treatment, even though more often than not, similar to chemotherapy and immunotherapy, success is marred by intrinsic or acquired resistance.

In this review, the term “epigenetics” refers to heritable, but potentially reversible, changes in gene expression or cellular phenotype that occur without alteration to the underlying base pair sequence [4]. Given the reversibility of epigenetic marks, which dynamically regulate gene expression, the nascent field of Episensitization [5,6] has emerged to address the treatment-resistance issue with chemo- and immune-therapies as a means of priming malignant cells to respond (or re-respond) to treatment with epigenetic inhibitors; by extension, the rationale for an episensitization strategy in radiotherapy distilled to a simple clinical syllogism (à la all men are mortal. Socrates is a man. Therefore, Socrates is mortal), is that (1) epigenetic inactivation of beneficial genes contributes to radioresistance (2) unlike genetic mutations, epigenetic alterations are reversible (3) ergo, conclusion, targeting epigenetic enzymes with small-molecule inhibitors should reverse non-responsiveness to radiotherapy.

The Merriam-Webster dictionary defines a syllogism as “a formal argument in logic that is formed by two statements and a conclusion which must be true if the two statements are true”. In this review, the syllogism is intended as a framing device to support a more detailed analysis of the aberrant role of epigenetic gene silencing in radioresistance and potential for therapeutic targeting to radioprime refractory tumors.

**Figure 1:**
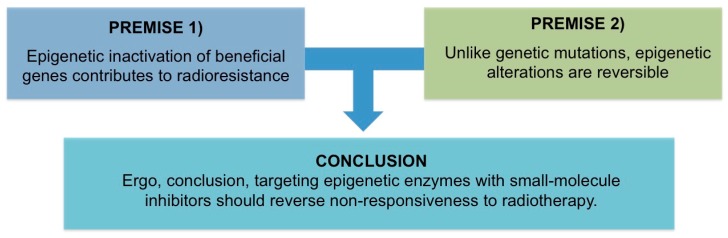
According to the conventions of a syllogism, if premise 1 and premise 2 are correct, then the conclusion is valid.

Each component of the syllogism, represented as a diagram in Figure 1, is reviewed below.

## 2. Premise #1: Epigenetic Inactivation of Beneficial Genes Contributes to Radioresistance

Based on preclinical and clinical evidence two major mechanisms of radioresistance exist and epigenetic alterations may involve both.

### 2.1. DNA Repair

**Figure 2:**
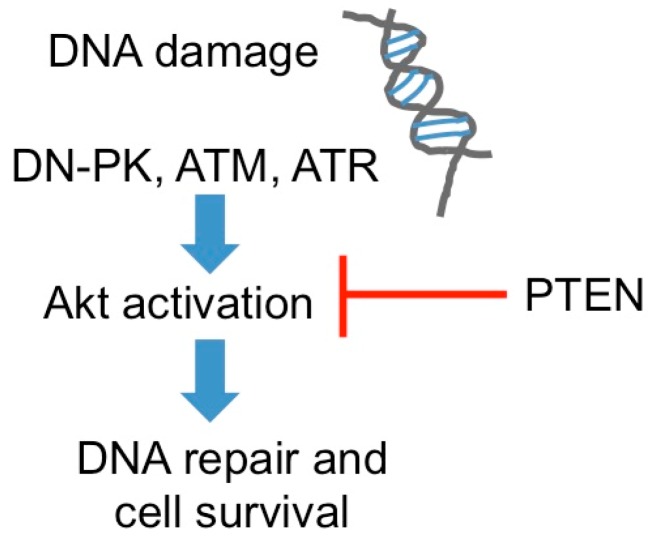
Molecular mechanisms and targets for Akt-mediated DNA repair. Akt can promote DNA repair and survival, leading to radioresistance.

Increased DNA recombinational repair of radiation-damaged DNA facilitates recovery of the cancer cells and contributes to a radioresistant phenotype. Conversely, DNA repair deficient cells are associated with radiation hypersensitivity [7]. The phosphatidylinositol 3-kinases (PI3Ks) and its functional subgroup, phosphatidylinositol 3-kinase-related protein kinases (PIKKs) [8,9], namely ataxia telangiectasia-mutated kinase (ATM) and ataxia telangiectasia and rad3-related kinase (ATR) are the main proteins that repair DNA double strand breaks (DSB) via nonhomologous end joining (NHEJ) and homologous recombination repair (HRR) [10]. Activated ATM kinase accelerates repair of radiation-induced DNA-DSB and, consequently, improves post-irradiation cell survival while inhibition of ATM correlates with the reversal of radioresistance [11]. Epigenetic silencing of phosphatase and tensin homolog (PTEN) on chromosome 10, a tumor suppressor gene that antagonizes the PI3K/AKT pathway [12] has been observed in several human cancers [13,14], which results in constitutive AKT activation, DNA repair and radioresistance (Figure 2). Epigenetic drugs also hold the potential to eradicate cancer progenitor cells [15].

### 2.2. Hypoxia

In a 1955 landmark study, Thomlinson and Gray [16] demonstrated that hypoxia, present in nearly one-third of all tumors [17], is pathognomonic for malignancy. While in normal tissues median oxygen concentrations range from 40 to 60 mmHg, tumors demonstrate pO_2_ fractions as low as ≤2.5 mmHg. By contrast, the lowest observable pO_2_ value in normal tissues is 12.5 mm Hg [18]. Low oxygen concentrations are definitively associated with radioresistance, tumor recurrence after radiation therapy, and poor outcomes [19]. The mechanism of hypoxic cell radioresistance is related to oxidative DNA damage from the generation of free radicals. In the absence of oxygen, hydrogen donation from sulfhydryl-containing (-SH) groups readily repairs the damage. In the presence of oxygen the damage is “fixed” or made permanent [20]. Thus, compared to well-oxygenated cells [21], hypoxic cells require an increased radiation dose (by a factor of 2–3) to achieve an equivalent level of cytotoxicity.

Epigenetic changes in normal tissue have been associated with adaptation to long-term transient hypoxia as experienced in ischemia and increased expression of the DNA methyltransferases 1 (DNMT1) and 3b (DNMT3b), directly linked to hypoxia-inducible factor 1α (HIF-1α) [22], may be important in the development of a fibrotic phenotype in the heart [23]. In a further example, the Jumonji-domain dioxygenases, a histone demethylase sub-group, are important factors that are upregulated during hypoxia [24] and in tumors, for example, colorectal cancer [25]. These, specifically Jumonji domain-containing 1a (JMJD1A) and 2b (JMJD2B), dynamically respond to hypoxia through histone methylation thus controlling the assembly of chromatin and thereby gene expression [26]. These factors often affect pathways that are critical to some cancers, for example JMJD1A regulates androgen receptor target genes [27]. Chronic hypoxia has been linked to global hypomethylation [28] resulting in genomic instability and leading to tumorigenesis [29]. Interestingly, these observations are in sharp contrast to the increase in global methylation in normal tissue as a consequence of hypoxia, suggesting that mechanisms involving HIF activation may be or greater importance in tumor but not tissue when exposed to low oxygen [30].

**Figure 3:**
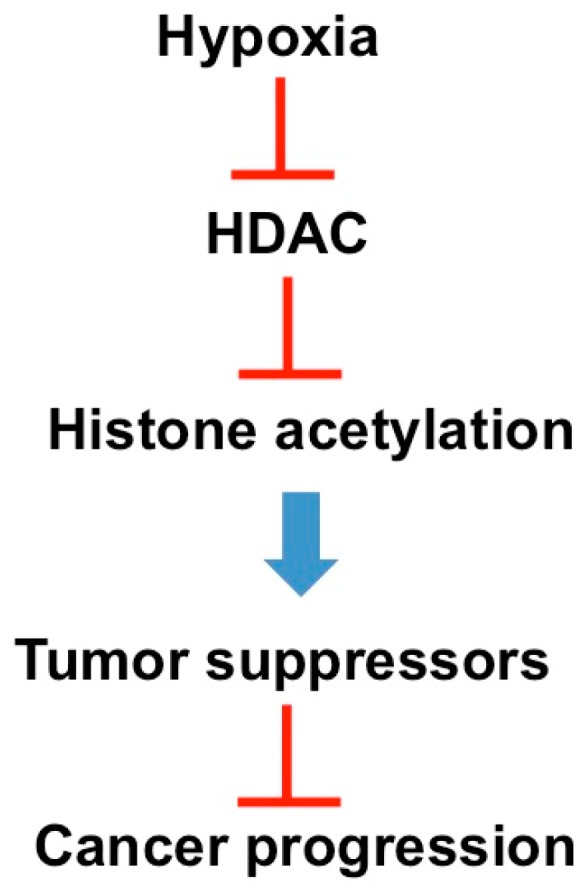
A simplified schematic of the effects of chronic hypoxia on the transcriptional repression of tumor suppressors such as p53.

Two types of hypoxia [31] have been recognized: (1) transient or acute (2) continuous or chronic. The former induces stabilization of the oxygen-sensitive subunit, HIF-1α, resulting in transcription of multiple angiogenesis-promoting target genes [32,33,34] including vascular endothelial growth factors, vascular endothelial growth factor receptors and erythropoietin. The latter is associated with transcriptional repression of tumor suppressors such as p53 via hyperdeacetylation [35] of chromatin, in particular, which correlates with increased aggressiveness and therapeutic resistance (Figure 3).

Although DNMTs have not been studied clinically as epigenetic agents that specifically target hypoxia, in vitro and in vivo inhibition of DNA methylation in the Von-Hippel Lindau tumor suppressor protein (VHL) promoter region by decitabine led to re-expression of VHL in clear cell renal carcinoma [36]. The accumulation of VHL mimics normoxic conditions under which VHL binds to HIF-1a enabling ubiquitination of HIF-1a and thereby its clearance. These data suggest that apparent reversal of the hypoxic response could be a potential clinical target for DNMTs.

Histone deacetylase (HDAC) inhibitors, particularly types I and II [37], can also influence DNA methylation [38] and reduce HIF transcription through a mechanism that may involve the inhibition of acetylation of the HIF transcription complex [39]. The inhibition of transcription of HIF-1a and HIF-2a through transactivation was not dependent on VHL, suggesting that these agents may have activity in tumors lacking VHL and may augment the hypoxia limiting activity of DNMTs when dosed in combination. The HDAC inhibitors romidepsin [40] and panobinostat [41] have been found to negatively regulate HIF and, by extension, angiogenesis [34], leading to increased chemosensitivity that is mediated perhaps, in part, through vascular normalization and improved therapeutic delivery.

Histone methylation has also been implicated in resistance to XRT. In particular, H3K27 methylation, highly expressed in recurrent glioblastoma after treatment with XRT, has been correlated with poor patient prognosis [42]. Indeed, radioresistant glioblastoma multiforme (GBM) cell populations were sensitized to XRT by Enhancer of Zeste Homolog 2 (EZH2) inhibitors [43].

## 3. Premise #2: Epigenetic Alterations Are Reversible

Epigenetics is on a continuum between genetic and metabolic mechanisms to alter gene expression. Genetic mechanisms such as mutations that involve direct changes to the DNA code itself, are heritable but relatively unsusceptible to exogenous influence, since mutation is a relatively rare event. Metabolic responses, which may initiate gene expression are responsive to exogenous influences, e.g., chemical substrates, but not heritable. Epigenetic modifications have characteristics of both metabolic and genetic mechanisms because, on the one hand, they are heritable and may persist after the exogenous influence is removed, but, on the other hand, they are also reversible. As defined earlier, epigenetic modifications refer to heritable changes in gene expression that occur without an alteration to the sequence or structure of DNA [4]. If the DNA code is comparable to a hard drive, then the epigenome is its operating system, which provides a selective readout of the information contained in the DNA sequence [44]. Unlike the permanence of genetic changes, epigenetic modifications are, in principle, reversible because no primary DNA sequence changes are implicated [45]. This unique property of reversibility raises the prospect that interventions which act on epigenetic regulators may overcome or even revert the resistant phenotype. The paradox of epigenetic effects is that they are, on the one hand, highly dynamic and subject to changes like metabolic responses and, on the other, stably conserved [46] like genetic mutations due to maintenance and inheritance mechanisms that propagate the epigenetic alterations across generations of cancer cells (Table 1). The heritable epigenome is DNA methylation-based while modifications to histone acetylation are more flexible [47]. Practically, the induction of stable changes in gene expression and the “memory” or persistence of this non-static epigenetic status quo means that the administration of epigenetic therapies may, in theory, precede (by weeks or months) different treatment modalities such as radiotherapy and/or chemotherapy to decrease the risk of concurrent and possibly overlapping toxicities.

The most studied mechanisms that affect the epigenome are DNA methylation and histone modification.

**Figure 4:**
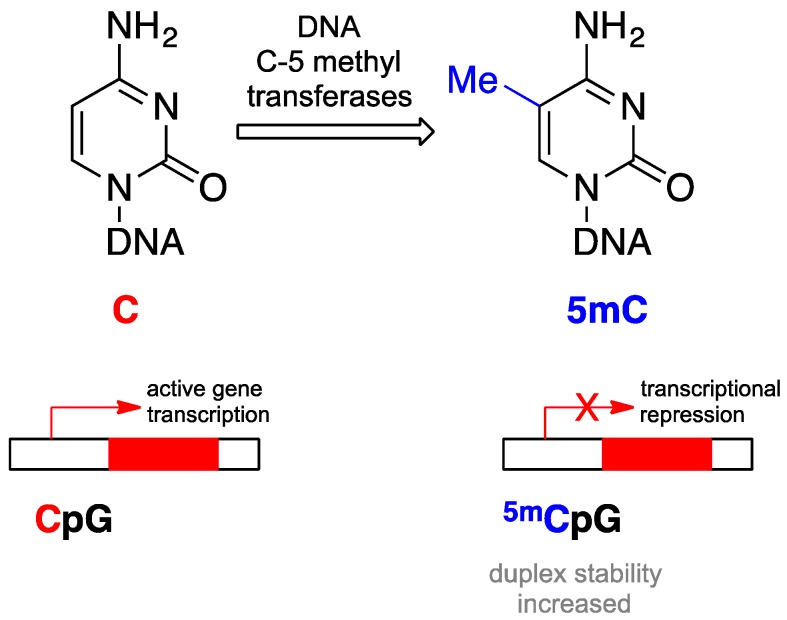
Schema demonstrating transcriptional repression with DNA methylation on cytosine.

DNA methylation involves the covalent addition of a methyl group to the 5′ position of cytosine, primarily cytosine-guanine or CpG dinucleotides [48]. The CpG dinucleotide pattern is relatively underrepresented in the mammalian genome but tends to cluster in short CpG-rich regions termed CpG islands (Figure 4) [48].

In the cancer cell, unlike the normal cell, most of the CpG dinucleotides at gene promoter regions are methylated [49], which is associated with transcriptional silencing. The mechanism of transcriptional silencing is presumably related to steric hindrance of the bulky methyl groups, which physically block the transcriptional machinery from accessing the DNA [50]. DNA methyltransferases (DNMTs) are the enzymes responsible for DNA methylation. As a maintenance methyltransferase, DNMT1 largely copies the DNA methylation pattern from the parent cell to the daughter cells, whereas the de novo methyltransferase proteins, DNMT3a and DNMT3b, generate new methylation marks in quiescent cells [51].

**Figure 5:**
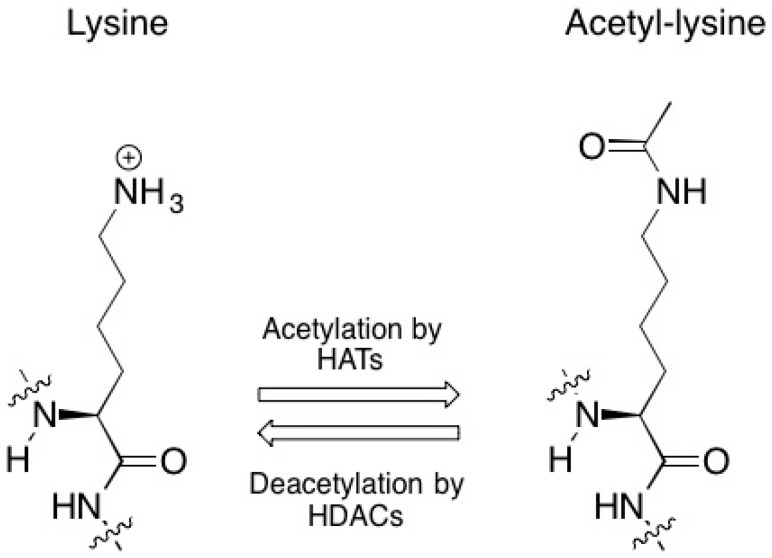
The enzymatic interconversion of lysine and acetyl-lysine.

Due to space limitations in the microscopic nucleus, the length of DNA or chromatin is compacted via ”spooling” around the outside of the histone octamer or nucleosome [52]. Posttranslational modifications from specific enzymes that include histone acetyltransferases, histone deacetylases, and histone methyltransferases influence whether the DNA tightly wrapped around the nucleosomes is accessible to the transcriptional machinery [53]. In effect, acetylation of lysine residues from histone acetyltransferases neutralizes the net positive charge of the histones, which, in turn, loosens its affinity for negatively charged chromatin and ultimately renders the DNA more accessible to the transcriptional machinery [54]. By the same token, histone deacetylases, which remove acetyl groups from lysine residues and thereby increase the electrostatic interactions between the DNA and the histones, prevent access of the transcriptional machinery and repress gene expression [55] (Figure 5).

Thus, in general, hyperacetylated regions of chromatin are transcriptionally active, while hypoacetylated chromatin is silent.

It is for these reasons that treatment with cytosine methylation and histone deacetylation inhibitors may lead to stable (i.e., transgenerational) re-expression of beneficial genes that have been silenced by cancer cells.

The duration of action of epigenetic agents is unknown and differs from targeted therapy. With targeted therapy, inhibition of key signaling enzymes leads to changes in signaling patterns that revert after the pressure of inhibition is withdrawn (or the tumor switches to a redundant pathway during treatment) leading to the development of resistance. It is commonly known that the action of epigenetic agents persists after treatment with an epigenetic agent is discontinued. It is therefore not certain how long epigenetic effects last, but these will depend on (1) the epigenetic event (e.g., DNA methylation versus histone acetylation), (2) the type of tumor and the location of the epigenetic modification, and (3) the pharmacodynamics of the epigenetic agent. For example, bromodomain inhibitors are designed to both bind and degrade the protein thus extending their duration of action. Thus radiosensitization with epigenetic agents opens a window of opportunity for the radiation oncologist to exploit in treating patients.

## 4. Discussion of Analysis: Targeting Epigenetic Enzymes with Small-Molecule Inhibitors *Should* Reverse Non-Responsiveness to Radiotherapy

The use of the verb “should” in italics above is intended to express expectation. With the caveat that this specific hypothesis requires testing and validation in prospective trials, the assumption is that epigenetic agents will reverse radioresistance based on preliminary evidence of clinical benefit in patients refractory to chemotherapy and immunotherapy. Indeed, on the premise that chemo-, immune- and radioresistance of cancer cells share common epigenetic mechanisms, scant but promising data from clinical trials demonstrating chemotherapy and immunotherapy priming is presented below.

### 4.1. Epigenetic Inhibitors as Chemotherapy Primers

In a Phase I/II study of 5-azacitidine and carboplatin 46% of patients with platinum-resistant or refractory ovarian cancer demonstrated durable responses and stable disease (median duration of therapy 7.5 months) [44]. In addition, an ongoing randomized Phase II trial (NCT02096354) called ROCKET with the experimental systemically nontoxic epigenetic agent, RRx-001, followed by irinotecan rechallenge on progression of RRx-001 has resulted in ”episensitization”, i.e., tumor resensitization by epigenetic mechanisms to irinotecan in multiple patients [5].

One general mechanism of sensitization to both chemotherapy and radiotherapy involves epigenetic restoration of silenced tumor suppressor genes such as p53 [56] and PTEN. Another mechanism of radio/chemosensitization is through blood vessel normalization, which enhances both oxygenation and drug delivery in tumors [57]. Due to the tortuosity and aberrancy of the tumor vasculature, alteration or “normalization” of the tumor vasculature also correlates with increased T cell infiltration.

There is extensive preclinical literature on chemosensitization through the action of epigenetic agents. Notable examples are combinations of a HDAC inhibitor with a TNF-related apoptosis-inducing ligand (TRAIL) receptor agonist [58], and how treatment with both histone deacetylase inhibitors and DNA methyl transferase 1 inhibitors can sensitize drug resistant ovarian cancer cells [59].

### 4.2. Epigenetic Inhibitors as Immunotherapy Primers

In addition to sensitization of chemotherapy, epigenetic agents (e.g., 5-azacytidine, 5-AZA) have been incorporated in a strategy to prime immunotherapy responses [60,61]. In 5 patients with non-small cell lung cancer (NSCLC) who received 5-azacytidine and entinostat prior to treatment with either anti-programmed death 1 (PD-1) or anti-PD-1 ligand 1 (PD-L1) antibodies, three complete responses and two durable stable diseases were observed [60]. Based on this demonstrated clinical benefit, a pretreatment study with azacitidine and entinostat or azacitidine alone prior to the PD-1 inhibitor nivolumab (NCT01928576) was initiated with the primary endpoint of overall response rate [62,63].

Another example of this immune priming strategy involves combination treatment with the experimental systemically non-toxic pan-epigenetic inhibitor, RRx-001, and the PD-1 inhibitor, nivolumab. Preliminary results from the first cohort of patients in a Phase I dose escalation study called PRIMETIME (NCT02518958) indicate promising safety and activity [64].

### 4.3. Epigenetic Inhibitors as Radiotherapy Primers

Clinical studies of epigenetic agents acting as radiotherapy primers are few and far between, however a Phase II study of the DNA de-methylator hydrazalazine and the HDAC inhibitor magnesium valproate was carried out in FIGO stage III cervical cancer patients (NCT00404326). The patients received a combination of hydralazine and magnesium valproate seven days before commencement of the combination therapy of cisplatin and radiation. Although this was a single arm study, and thus comparisons are difficult to make, preliminary results suggested that the combination was effective as all evaluable patients achieved clinical complete response during the external radiation compared to the anticipated 75% rate in historical controls [65].

## 5. Conclusions

Once almost exclusively considered a gene-centric or genomic disease, evidence indicates that pathognomonic epigenetic alterations are also a hallmark of cancer [66]. These epigenetic alterations serve as a mechanism for the cancer cell to turn off the transcription of genes that mediate susceptibility and therapy response.

Of the manifold challenges in oncology including non-selectivity, clinical toxicity and the heterogeneity of response, the one that is perhaps the most pressing, persistent and pervasive is the appearance of resistance, either intrinsic or acquired, which casts its long and baleful shadow [67] over all treatment modalities, including radiotherapy. Radioresistance is particularly insidious because it is currently impossible a priori to predict tumor response, and radiotherapy carries with it the risk of acute as well as chronic toxicities which may manifest months or even years later [68]. Given this potential for long-term harm, several authors have called into question the need for routine RT in indications [69] such as locally advanced breast cancer [70] where, despite an increased risk of cardiac toxicity, secondary cancers, pneumonitis, lymphedema and radiation-induced fatigue [71], and no randomized evidence to demonstrate which patients are likely to significantly benefit, chest wall irradiation is considered standard of care [72]. In the absence of a predictive test or biomarker, epigenetic agents, which relieve transcriptional repression, thereby turning on silenced regulators such as tumor suppressor genes, may a fortiori stack the deck in favor of a successful outcome. Of course, while the 3-D dynamics of the tumor are manifestly too complex to reduce to a one-dimensional epigenetic premise, transcriptional dysregulation and the abnormal epigenetic programming that underlies this dysregulation has emerged as a unifying theme in therapeutic resistance, whatever the modality [73].

As described by Strauss and Figg in the Lancet Oncology [74], the nascent field of Episensitization is at the forefront of a broadly applicable strategy to reverse resistance and prime tumor responses to chemotherapies and immunotherapies in the clinical trial setting with agents such as decitabine, entinostat and RRx-001. Epigenetic priming is perhaps even more likely to improve responses in a radiotherapy context given that HDAC inhibitors [75], decitabine [76], and RRx-001 [77], in particular, all induce oxidative stress, which, in turn, sensitizes cancer cells to the effects of ionizing radiation [78].

Radiosensitization is complementary to a radio-priming strategy since both, in theory, increase the activity of a given dose of ionizing radiation. However, unlike epigenetic radiosensitization [79], which implies concurrent dosing with RT and increases the risk of overlapping adverse events, radio-priming may involve a post-treatment “washout period” of days to weeks with the epigenetic agent to minimize combined toxicities, since the objective, subtly different from a radiosensitizing approach, is to stably reshape the gene expression pattern of the tumor, thereby enhancing its susceptibility to subsequent treatment. Transcriptional modifications due to DNMT or HDAC inhibitor therapy may “carryover” from one sequential treatment period to the next because, despite their potential reversibility, epigenetic marks are stably propagated during mitosis, leading in some cases to long-term retention of particular gene expression patterns [80,81], akin to a kind of epigenetic memory. In other words, reprogramming the cancer cell with DNMT or HDAC inhibitors to a “normal-like” state through the forced re-expression of silenced genes has to the potential to provide a durable and stable antitumor “memory” response [82].

This “carryover effect” is assumed to be absent or negligible with a traditional concurrent chemoradiation paradigm. Indeed, historically, the main criticism of radiosensitizers, as a whole, has been the narrow benefit to toxicity ratio: the long and checkered history of radiosensitization includes a laundry list of failures, from the nitroimidazoles and halogenated pyrimidines to cisplatin and 5-fluorouracil (5-FU) as potentiators of radiation toxicity [20,83]. By contrast, pretreatment with reactive oxygen species (ROS)-inducing epigenetic agents may not only limit overlapping toxicities due to separate “time windows” of administration but also radioprotect normal tissues; exposure to oxidative stress has the potential to precondition these tissues against further oxidative stress from RT in the same way that ischemic hearts may adapt to the oxidant injury from ischemia reperfusion [84]. In fact, RRx-001 and the organic arsenic compound darinaparsin, both experimental ROS-generating radiosensitizers, are also associated with protection of normal intestinal crypt epithelial cells from clonogenic death after ionizing radiation [77,85,86].

As data from clinical studies mature and, hopefully, continue to validate episensitization as an important anti-resistance strategy for chemotherapies and immunotherapies, it should extend its boundaries to include radiotherapy so that most, if not all, of the sixty percent of cancer patients (that is, the sixty percenters) who receive it at one time during the course of therapy derive benefit.

## Figures and Tables

**Table 1:** Reversibility of gene expression changes.

Influence Gene Expression					
Change Type	Susceptible to Induced Change	Reproducible	Reversible	Persistent after Stressor Is Removed	Heritable
Genetic	Limited	Partially	No	Yes	Yes
Epigenetic	Yes	Yes	Yes	Yes	Yes
Metabolic	Yes	Yes	Yes	No	No
